# Examining social class as it relates to heuristics women use to determine the trustworthiness of information regarding the link between alcohol and breast cancer risk

**DOI:** 10.1371/journal.pone.0270936

**Published:** 2022-09-12

**Authors:** Samantha B. Meyer, Belinda Lunnay, Megan Warin, Kristen Foley, Ian N. Olver, Carlene Wilson, Sara Macdonald S., Paul R. Ward

**Affiliations:** 1 School of Public Health Sciences, University of Waterloo, Waterloo, ON, Canada; 2 Research Centre on Public Health, Equity and Human Flourishing, Torrens University Australia, Adelaide, South Australia, Australia; 3 School of Social Sciences, University of Adelaide, Adelaide, South Australia, Australia; 4 School of Psychology, Faculty of Health and Medical Sciences, University of Adelaide, Adelaide, South Australia, Australia; 5 School of Psychology and Public Health and the Olivia Newton-John Cancer Wellness and Research Centre at Austin Health, La Trobe University, Melbourne, Victoria, Australia; 6 Institute of Health & Wellbeing, University of Glasgow, Glasgow, Scotland; CNR, ITALY

## Abstract

**Background:**

High rates of alcohol consumption by midlife women, despite the documented risks associated with breast cancer, varies according to social class. However, we know little about how to develop equitable messaging regarding breast cancer prevention that takes into consideration class differences in the receipt and use of such information.

**Objective:**

To explore the heuristics used by women with different (inequitable) life chances to determine the trustworthiness of information regarding alcohol as a modifiable risk factor for breast cancer risk.

**Methods and materials:**

Interviews were conducted with 50 midlife (aged 45–64) women living in South Australia, diversified by self-reported alcohol consumption and social class. Women were asked to describe where they sought health information, how they accessed information specific to breast cancer risk as it relates to alcohol, and how they determined whether (or not) such information was trustworthy. De-identified transcripts were analysed following a three-step progressive method with the aim of identifying how women of varying life chances determine the trustworthiness of alcohol and breast cancer risk information. Three heuristics were used by women: (1) consideration of whose interests are being served; (2) engagement with ‘common sense’; and (3) evaluating the credibility of the message and messenger. Embedded within each heuristic are notable class-based distinctions.

**Conclusions:**

More equitable provision of cancer prevention messaging might consider how social class shapes the reception and acceptance of risk information. Class should be considered in the development and tailoring of messages as the trustworthiness of organizations behind public health messaging cannot be assumed.

## Introduction

Primary breast cancer prevention typically relies on increasing women’s awareness of modifiable risk factors [1, 2]. Alcohol is a modifiable risk factor that has received less attention than others in Australian campaigns aimed at breast cancer prevention [3], despite clear evidence for its breast cancer-causing properties [4–6]. Epidemiological studies have demonstrated that even consumption at low levels, previously considered ‘safe’, elevates a woman’s breast cancer risk [7–10]. As such, communication regarding alcohol as a risk factor has been identified as a critical public health priority [11].

Despite prioritization of alcohol as a risk factor for breast cancer in communication efforts, research conducted in Australia suggests that women in midlife are largely unaware that alcohol is a breast cancer risk factor [3] or they choose to accept the risk given the important role it plays in their lives (e.g., as a source of pleasure or a coping mechanism) [12]. Widely accepted social norms surrounding alcohol in Australia [3, 13] and an alcohol industry actively working against health messages about alcohol-related cancer in particular [14, 15] further hamper risk communication efforts.

To complicate communication efforts even more, women are not a homogenous group and as such, messaging cannot be uniform. Central to the current paper, research with midlife women (those aged 45 to 64 years) demonstrates that the level of alcohol consumption and the reasons women provide for consuming it varies according to social class [16, 17]. These class-based differences in consumption suggest that the sources of information that women look to, and trust, regarding breast cancer risk may also vary according to the social, cultural and economic resources available to women. As such, social inequities in breast cancer risk, as they relate to cancer prevention efforts, are a concern.

There is a vast literature documenting social class as a predictor of health behaviours. Indeed, research has shown that social class (and other social determinants of health) impacts cancer information seeking [18]. However, studies of social class and health behaviour largely focus on the unhealthy behaviours of lower social class, and simply note that people in upper social classes have healthier lifestyles [19]. Further, social class is often measured based on income and education, despite the suggestion that engagement in prevention behaviours may be more nuanced than considerations of education and income [19]. Rather, differences in engagement between social classes may also be due to “socialisation, norms, health control beliefs and health knowledge that develop through educational systems” [19] (p. 24). This literature was critical for the present study whereby women were assigned to different class groupings based on their differential access to economic, social and cultural capital [20–22] that shapes their life chances. Further, we explore the ways in which social capital might also intersect with trust, providing a more nuanced understanding of the heuristics by which women engage with and/or accept prevention information.

While some scholars have argued that social class is no longer relevant to understanding the distribution of contemporary risk [23], this has been vehemently disputed—for a summation literature see Curran [24]. In fact, following a logic that life chances (and life choices) are socially structured and can result in differing circumstances of equity and advantage, class (and its intersections with gender, race, among other constructs) maintains its position as a key player. Curran argues that “growing risk ‘reinforces’ rather than ‘transforms’ the logic of social distribution” [25]. That is, growing risk furthers the social divide, rather than neutralizing it. Indeed, there is a social gradient in outcomes across the breast cancer incidence, diagnosis, treatment, survival and mortality in Australia, though the strength and direction of this relationship varies along the continuum [26]. Here we focus on the gradient as it relates to prevention.

We adopt Curran’s logic and explore how the alcohol-breast cancer risk message is received by women with different (inequitable) life chances, according to social class. We draw on data collected as part of a larger study investigating consumption of alcohol during midlife in a sample of Australian women from differing social classes—exploring their knowledge and perceptions of their own breast cancer risk. We do so with a recognition that social class is one factor that intersects with inequitable life changes, and thus trust and experiences of health, but note that intersecting social locations (e.g., ethnicity, race, sexual orientation) are beyond the scope of this paper. Our goal is to extend our understanding of how public health messaging might reflect class-based differences as an avenue for working towards greater equity in breast cancer prevention efforts.

### Alcohol, breast cancer risk and trust

Breast cancer is the most common cancer affecting women in Australia [27]. Despite research demonstrating that alcohol is a risk factor for breast cancer [7, 9, 28], the regular consumption of alcohol is widespread in Australia, with roughly 17% of Australians consuming alcohol at levels that increase their risk of harm over their lifetime [29]. Moreover, alcohol consumption levels among Australian women during midlife remain higher than other female age groups [30–33]. Increasing knowledge of the link between alcohol and breast cancer risk is an important first step to reduce breast cancer prevalence [34]. Public health professionals continue to design frameworks that guide risk messaging with the aim of reducing alcohol consumption to improve health outcomes [35–37]. However, it is well-established that if these messages, or the messengers, are not trusted, efforts are likely to be futile, with empirical support showing how critical trust is for the acceptance of preventative health information [38–44]. When people trust a source of information, it shapes their decision to pay attention to information from that source, and the likelihood that they will act on the information obtained from that source [45].

At a population level, women look beyond formal institutions to various sources for breast cancer information, including the media, friends and family [46], the internet [47] and also web-based risk assessment tools [48]. The scant literature looking at trust and breast cancer prevention messaging is focused on tailored communication strategies that incorporate trust for the purpose of communicating risk to equity-deserving populations specifically (e.g. populations with disparities in access due to cultural or language barriers) [49, 50], or trust in sources of information among women following a breast cancer diagnosis (rather than prevention) [51–53]. Research documenting how people more generally choose source(s) of health information, how they assess its trustworthiness [54], and whether these processes vary by class however, is limited; and even more so when we consider alcohol specifically. We do know, however, that trust is classed, with evidence of lower levels of generalized trust among lower social classes, as the costs of perceived betrayal of trust are greater amongst individuals without access to material or financial security [55]. To respond to these gaps, we draw on a relational trust framework to explore how individuals in different social classes make decisions about what is, or is not, trustworthy.

### A relational trust framework

Trust is an outcome of a valued relationship. Calnan, Williams [56] have described trust as existing when the “trustor” (in this case, midlife women) have positive perceptions of the competence, knowledge and skills of the “trustee” (the information source, or those responsible for the message), and anticipate that they will act with beneficence, fairness and integrity. Trust is therefore said to not be a solely individual phenomenon based on an individual’s perceptions but rather, something that is founded in and developed from and within social relations [57].

There has been a proliferation of breast cancer risk messaging and indeed, misinformation, available to women regarding breast cancer risk [58]. In the context of messaging about the link between alcohol and breast cancer then, the trustee may be a healthcare provider, someone posting on a personal social media feed, traditional media, a person’s doctor or other health professional, a non-government organization (NGO) or the State or Federal Government. Whether the source of information is an individual or an organization, if trusted, the expectation by women would be that the information or message being conveyed is in the adopter’s best interest and grounded in truth.

The choice to trust is complex and consists of both a cognitive component (e.g. an assessment of the knowledge of the messenger) and an affective component [59, 60]. Trust also requires some element of risk-taking (i.e., trusting without certainty); it is an active decision. If risk is not considered, one is said to have confidence, rather than trust [61]. This may be particularly relevant where health message acceptance hinges on risk, as it is the case for preventing alcohol-related breast cancer. Although it is certain that alcohol has cancer forming properties [4–6], it is not certain that consuming it will lead to a breast cancer diagnosis, as there is variability in risk in accordance with women’s unique physiology. In this regard, alcohol is not unlike various other modifiable risk factors for cancer that we act upon (e.g., smoking tobacco, physical inactivity). The inability to prove one will most certainly develop cancer because of consuming alcohol is used by the alcohol industry to falsely suggest that it is not possible to act on the evidence that alcohol causes cancer. We are therefore asking women to trust a public health message and accept the risk(s) of giving up alcohol (e.g., forgoing pleasure or a coping mechanism) without knowing if continuing to consume would indeed lead to breast cancer. Further, even if consumption is decreased, they may still develop breast cancer.

Unique in the present analyses are considerations of the social relations that occur as a result of social class position. Drawing on a relational model of trust, we propose two key aims: (1) to identify in whom and in what women trust about breast cancer risk (in particular, alcohol as a modifiable risk factor) and how they determine the trustworthiness of these sources; and (2) to identify how the patterning of processes women use to make decisions about what is trustworthy is ‘classed’—that is, it is embodied and practiced within a cultural reference system that shapes women’s ability to trust and be trusting of information. The information generated might be used to develop tailored, class-based prevention messaging to work towards greater equity in breast cancer prevention efforts.

## Materials and methods

The study had full ethical approval from Flinders University Human Research Ethics Committee (Project 1938). Participants provided informed written consent and pseudonyms are used herein to preserve anonymity. Interviews were undertaken with 50 women aged 45–64 years (termed ‘midlife’) living in South Australia. Aspects of these interviews that pertain to women’s perceptions of alcohol as a risk factor for breast cancer have been published elsewhere [3, 62]. In the analysis presented, we specifically explored the role of women’s social class–their differing access to economic, social and cultural capital [20–22] that shapes their life chances–as it relates to trust in sources of information regarding alcohol-related breast cancer risk. Participants represented a diversity of self-reported alcohol consumption levels, but most described themselves as ‘light’ or ‘moderate drinkers’. Some ‘occasional’ and ‘heavy’ drinkers were also recruited in order to achieve maximum variation sampling [63].

To ascertain the social class of individual women, we utilised questions from the 2011 Great British Class Survey [21] which had been adapted for use in Australia [16, 22, 64]. The breakdown of the sample is provided in Table 1. Full details of the sample and recruitment process are described elsewhere [17, 62].

**Table 1:** Social class groups: Class labels and compositions of capitals comprising each group.

Class characteristics
**Established working class** (n = 14)–Low economic, social and cultural capital
**Established middle class** (n = 9)–Medium economic, social and cultural capital
**Mobile middle class** (n = 8)–High economic and social capital; low cultural capital.
**Emerging affluent class** (n = 14)–Medium/high economic capital (high incomes but low property/wealth/assets), high social capital, medium/high cultural capital.
**Established affluent** (n = 5)–High economic, social and cultural capital

### Interview questions and approach

Researchers BL and KF both female and aged in their 30s conducted the interviews. Each interview began by collecting a ‘life history’ from women, which included aspects of their family life and upbringing and the impact on their knowledge of health information. Additionally, they provided a description of any health information seeking behaviour and knowledge of risk factors for breast cancer, including alcohol. Women described: where they sought health information, how they accessed information specific to breast cancer risk as it relates to alcohol, and how they determined whether (or not) such information was trustworthy. Following the logic that ‘naturalistic’ exchanges are useful for engaging with participants’ subjectivities [65], participants were encouraged to expand on the questions within the remit of topics of research interest. Interviews were audio-recorded, transcribed verbatim and de-identified.

### Data analysis

De-identified transcripts were analysed following a three-step progressive method of 1) pre-coding, 2) conceptual and thematic categorisation and 3) theoretical categorisation, a process for synthesising social theory within qualitative analysis [66]. Transcripts were manually and inductively pre-coded before electronic copies were imported to NVivo QSR (v12) data analysis software [67]. Using NVivo, a preliminary coding framework was developed based on a combination of pre-coding and the team’s expertise in social theories of trust [68–71]. At this stage, the research team co-coded a selection of the transcripts for reliability testing and to determine additional codes to add to the preliminary coding framework. Four of the authors (BL, KF, PRW, MW) independently pre-coded 6 transcripts against the framework. The team conferenced to compare coding and subsequently revised the codes and modified the framework—a strategy to achieve interpretive validity—which then guided coding of all transcripts (n = 50). Finally, using a combination of inductive and deductive logic [72] codes were collapsed into broader categories and themes, creating a hierarchical coding framework and linkages between the categories and concepts were identified.

All codes relevant to the present analysis were then reviewed in detail by author SBM who identified the ‘heuristics’ with which women engage to judge the trustworthiness of information about breast cancer risk. In consultation with author PRW an expert in trust theory, data were analysed in consideration of the relational trust framework. Following the framework analysis, we looked within findings to identify differences between the social class categories. We also ran matrix queries of content coded at key themes alongside class variables. This method of quantifying the coding identified classed patterns in the coding (i.e., transcripts of interviews with women in social class groups where themes were more common) and identified areas for closer scrutiny.

## Results

Data are organized into three sections that describe three heuristics women engage with to determine the trustworthiness of alcohol and breast cancer risk information: (1) consideration of whose interests are being served; (2) ‘common sense’; and (3) evaluating the credibility of the message and messenger. Embedded within our discussion of each heuristic are notable class-based differences documented throughout our analysis.

### Heuristic 1: Consideration of whose interests are being served by the messaging

Consideration of whose interests are being served was a frequent theme throughout our interviews. Women in this study, unprompted, spoke frequently of the varied sources and content of the information about alcohol and cancer to which they were exposed. Many spoke to their questioning of the interests being served and consequently the trustworthiness of the message. For example, when asked where she would look for health information, Bernadette (Emerging affluent class) responded: “Anybody who hasn’t got money to make, or an axe to grind.” She then went on to speak about having trust in social institutions (using universities as one example) because they are “in the business of public health.” She contrasted this with “big pharma” and “the bad guys like Monsanto what they get up to, and the stuff they do with GM crops” noting the huge money to be gained; again, relating the discussion to competing institutional objectives–making money versus supporting public health outcomes.

The word ‘commercialised’ was used several times in reference to information about health more generally. Both Anna (Emerging affluent class) and Michelle (Established middle class) discussed questioning information conveyed on traditional and social media (e.g., Facebook or Instagram feed). Women drew on examples beyond breast cancer to highlight the importance of questioning information across various sources. Michelle went so far as to differentiate between mainstream media sources as being more (the ABC, government subsidised media company) or less (Murdoch Press) credible.

“It’s all commercialised and everything else, and I know that. And it’s not just him, it’s all the other commercial stations as well; they give you this information and you know that there’s somebody else backing it up.” Michelle, Established middle class

Lois (Established middle class) also spoke about her approach to determining whether information was trustworthy, particularly if it came from an unknown or unfamiliar organization. She described:

“I think a lot of it, you base on, well first of all you just look at the tone of the way they wrote about things, you’d probably, if you thought it’d been sensationalized, or if they were axe-grinding…” Lois, Established middle class

She went on to describe the features of messages pushing an agenda and how she evaluates information, again largely pointing to balance and whose point of view is privileged.

“It’s like, look at, you just listen to it, see if they’re on, going to be harping on a certain theme, maybe without having any basis for saying so, if they’re trying to push a certain point of view without having any basis or back-up for that. Rather than someone being even-minded about, you know, even-handed about things. So, I mean the way people write things, you can often pick that up. But it’s also where the… what sources they site, I’d certainly be interested in that.” Lois, Established middle class

Similarly, Laureen (Emerging affluent class) explained how she is more likely to take note of messaging that is not in the form of an advertisement. For her, the commercial nature of the source helps to form a line between what is trustworthy, or not. In this excerpt Laureen refers to being ‘even-minded and even-handed about things’ which also suggests that messages of moderation (rather than complete abstention) are perhaps important to the way people view information.

“I’ll always consider things in the media. You know, even research things, and ads and those kinds of things, I will query where does the information come from and why is this person telling me this? I suppose I believe something that is occurring on the ABC, probably somewhat less sceptically than a commercial radio or TV, purely and simply because of the less commercial nature of it. I do always like to see ‘you know, this information is provided by such and such a research organization or such and such a product or chemical product or a women’s health organization’. I’d be more inclined to take notice of a women’s health organization promotion and/or a breast cancer support organization than some media outlet that’s purely and simply news.com, just because of advertising. I don’t–I’m not a big fan of advertising and a lot of the time if I see something advertised I’ll be averse to it rather than inclined to take it on… Sometimes and mostly, I would say. I suppose I do–I’d rather a personal story. I’d rather something biographical than advertising a product or produced by somebody who’s got something to sell, if that makes sense?” Laureen, Emerging affluent class

Laureen (Emerging affluent class) went on to state that in approaching information she asks “what’s the motivation of the person who’s giving me this information? Is it information for information’s sake or is it trying to flog something?”

When asked how she decides if information is trustworthy, Anna (Emerging affluent class) also spoke of methods for deciphering what is trustworthy information, speaking again to the ‘red flag’ raised if revenue is to be gained by a private organization and those promoting the message. Notable in her words below is the importance she places on the reputation of the institution that is behind a message. Here, the already trusted organization acts as signals that the information under this banner is trustworthy, thus facilitating ease in accepting information without further evaluation.

“I don’t know. I think the more often you see something it’s like, you know–it does take a few messages probably because there’s so often stuff that says ‘eat more of this then you’ll be healthier’ and ten years later ‘don’t eat any of that because it’s [not a thing]’. ‘Do this’ and then a bit later ‘oh no, don’t do that’. I think if there’s something that’s actually researched and has–and usually if like it says Flinders Medical Centre or it has the research name behind it that you recognise from another hospital, Monash or whatever, then you do–I think you probably do tend to believe it and the more often you’ll–if you see that someone else is quoting the research from that base you kind of go ‘oh, okay so they think it’s correct and these people support it and those people support it so it means it’s ridgy didge [authentic] and not just someone doing a paleo diet and earning lots of money’. Like the wellness blog, didn’t believe her.” Anna, Emerging affluent class

The final quote we present in this section illustrates a means for building trust, identified by participant Sandy (Mobile middle class). Until she saw the advertisement for our study, she was unaware of the link between breast cancer and alcohol. However, armed with the knowledge that some government funded public health authorities were trying to reduce alcohol consumption, she looked at the government favourably given her awareness that for the government, reduced consumption means reduced revenue. She noted:

“But I didn’t realise there was a link. And I went on and interrogated it after that, because I do enjoy a glass of wine, and I wondered, what am I knowingly getting into here. And to understand how alcohol affects your body, in terms of it increases the estrogen levels, and so that has a link to breast cancer. Which I found interesting to know. Not all women want to know that level of detail, and certainly, for this study, we’re interested to understand what women trust. And I speak to women, like I said, from all walks of life, and lots of women don’t trust the message, they think it’s conspiratorial, they think the government’s out to take away from them something that they enjoy. But you flip it on the other side, why would the government want to stop, reduce alcohol consumption and - - - … When they make money out of it?” Sandy, Mobile middle class

Across the data, it was largely women in the middle- and upper-class positions that questioned the validity of sources of information about breast cancer prevention through their own personal research.

### Heuristic 2: Engaging with ‘common sense’, ‘a pinch of salt’, ‘a gut-feeling’

Women frequently cited using / engaging with ‘common sense’ or a ‘gut feeling’ as a heuristic for weeding through the vast quantities of information about breast cancer risk to decipher what is trustworthy. They also spoke of interpreting messages with ‘a grain of salt’, indicating a level of scepticism.

As noted above, Sandy (Mobile middle class) learned of the link between breast cancer and alcohol through participating in the research study reported here. When reflecting on whether she trusted risk information, she reasoned that, given alcohol is problematic for health in other ways, there was some logic to its link to breast cancer. She described using her ‘common sense’ as a heuristic to help judge ‘new’ information she received.

“Yeah. So I guess that’s it. I haven’t seen any of the science, but I’m sure, if somebody said that there was science to support it, I’d go, yep, yep, that’s fine. And like I say, inherently, I would think that, well, alcohol consumption would–it’s the same thing, healthy lifestyle, if you don’t have a healthy lifestyle it’s going to increase your risk of a whole lot of things. So to me, it makes inherent sense, in that sort of manner as well too. So it’s about then looking at those risks, and then thinking, well, what’s the risk for me.” Sandy, Mobile middle class

The excerpt from our interview with Rebecca (Established middle class) seems to suggest that despite acknowledging the ‘sense’ in health information, this might not necessarily translate to a change in behaviour. Rather there is a process of weighing up the information against personal risk perceptions. Rebecca (Established middle class) indicated that widespread recognition of alcohol as an indulgence also confirmed the acceptability of the alcohol-breast cancer link, especially when the message was conveyed by a trustworthy source:

“I trust the Cancer Council to say the facts really. Whether I like them or not is a different–it’s like ‘what do you mean, sitting on the couch drinking half a bottle of wine is not good for me?” Rebecca, Established middle class

The idea that the association between breast cancer and alcohol is not surprising helps to explain why women in the middle or affluent classes, often stated that they drew on ‘common sense’ when looking to identify trustworthy information about breast cancer and health information broadly. For example, Abigail (Mobile middle class) suggested:

“I think sometimes the more information comes out or the more it’s repeated the more it becomes common knowledge for people rather than easily dismissed.” Abigail, Mobile middle class

Given the pervasive nature of breast cancer risk information promulgated in advertising campaigns and awareness raising events, it was unsurprising that women also spoke of picking up on bits of information about breast cancer risk over time. Drawing on their existing knowledge seemed to allow women to accept something as a ‘given’ or alternatively, to approach it with caution or some level of scepticism, serving as a catalyst for a decision about trustworthiness. Kimberly (Emerging affluent class) spoke of her foundations for judging materials as trustworthy based on the medium through which it is communicated. She viewed the organizational origins of a website as an indicator of the quality and reliability of the information it contains and expressed doubt about information promulgated on social media:

*Interviewer: So, the information that you’re grabbing off Google, do you know the source?*

Kimberly (Emerging affluent class): Usually I go to one that’s.org or.edu or you know, I don’t go to just.com or whatever. And in fact, there is a health site which, I can’t think what the name of it is now, but health, no, I can’t think. But I usually go to one where it’s a reputable…

*Interviewer: Yeah. If there was to be some information on social media, if that was a way that information was shared, what would you think of that?*

Kimberly (Emerging affluent class): I’d probably take it with a pinch of salt. I wouldn’t necessarily take it as being correct…No. I wouldn’t use that. You know, they have some of these ads on there that show you if you eat this what will happen to you. And I think, “What a load of rubbish.” I don’t look at any of them.

Among middle class participants, common sense also played a role in women’s readiness to accept advice. For example, Isabelle (Established working class) stated:

“…well you know like food, exercise, breathing good air. All that sort of thing. Is just plain good sense. Whereas injecting milkshakes, or whatever, vitamin shakes, or whatever, into your veins, to me, I think it just comes down to common sense…” Isabelle, Established working class (formerly established middle class before a divorce)

The expressions trusting your ‘gut’ and ‘gut feeling’ were also used to explain women’s trust.

“I don’t know. I guess it’s a gut feeling, isn’t it? I wonder if it’s a–if you’ve got some sort of education, I’m thinking, and some sort of knowledge I think your gut feeling will go ‘that’s rubbish’ or ‘that’s not enough research done on that’ but I would never look it up or anything. Others will always stick in your head I think with the ‘yeah, they’ve been working on that for a long time’ or yeah, that’s quite possible’ and so I think that’s how you work–yeah.” Ashleigh, Established middle class

For some, this gut feeling was prompted by signals within the information in question; for example, Shelley (Emerging affluent class) described considering the study design informing the information being provided, as well as the individuals who conducted the research.

“A lot of it’s gut feeling. You look at the research and you’d say ‘well, they’ve had a sample size of, you know, 100 women in, you know, one particular socioeconomic group’. Well, obviously that’s not going to be a broad selection. You know, I’d really discount that. If you look–they’ve done a lot of research on–you know, over a number of years in, you know, varying age groups, well, that seems to be a valid study and I’d take more notice of that than one that was, you know, some fly by night person, you know, that came up with some quacky sort of idea that–so I’d look in depth at both and make my decisions based, I think, on facts.” Shelley, Emerging affluent class

It was most frequently middle or affluent class women that spoke of using common sense, likely relating to the various forms of capital (e.g., social, cultural) they have to draw on in deciding what was ‘common’ in their social location. Women who identified as working class were more likely to describe judging information with a ‘pinch of salt’. They indicated starting from a position of scepticism and only accepted information as trustworthy over time. Tricia (Established working class) said:

“I do question quite a lot because I do think the media play it up… I tend to like to think it’s a bit of–you know, like I take it with a pinch of salt. I like to see like clinical trials and things like that, not that I can always understand them but I want to see evidence based, not some hype. So if I’m taking it [health information] from what some of those funny magazines–you know, I’ll take that with a pinch of salt. Like Kate [Middleton] has been pregnant 20 times according to them [the magazine] and she’s only just having her third baby w–ereas if I read it in a more upmarket sort of magazine I might put a more–yeah, just accept it more than if it is in one of those sort of wishy washy magazines.” Tricia, Established working class

### Heuristic 3: Evaluating the credibility of the message and source when making the decision to trust

Women were found to draw on ‘markers’ or signals of trustworthiness from the sources of information rather than focusing solely on the volume or content within the information. For example, when speaking about why they trust certain information above others, women were quick to reach for language that indicated a marker of trust (which was related to an institution, entity or individual) and it followed that trust in the alcohol and breast cancer message was based on this marker. Markers of trust included the message being based on ‘science’ or ‘research’, or as ‘credible’. Rebecca (Established middle class) noted that for information to be trusted it needs to be ‘verified’ and scientific, noting her ability to understand the research:

“I’ll go to government sites so, you know, health sites that are not Doctor Google with everyone–- with every quack being on there. I’ll make sure that this is a verified thing, yeah, because you just hear of people being duped into some dodgy stuff that you–you know ‘take these pills’ and it’ll be like they’re no good at all. It’s like, you know, have they had that trial? Have they been through human trials or have they just fed them to rats and go ‘oh yeah, that’s good’ or not even done that? I must admit I’m very ‘okay, so what’s the science that’s going to back this up?’ I mean even when I was making my decision about what treatment to take I read the scientific papers to go ‘okay, what does this mean?’” Rebecca, Established middle class

Lois (Established middle class) spoke of the need for her information from “well-founded health shows which are run by well-qualified people”. For her, well founded and qualified depended on the information being promoted coming from “orthodox science”. Similarly, Joanne (Established working class) said:

“Well, I know there’s been various research done but I have to admit I tend to be rather sceptical about certain research because my understanding and experience and things I’m aware of is that it often depends on how it’s done. It depends on how it’s analysed and things can be found that really say ‘oh, yes, this is what’–and then someone will come along and ‘no, it’s not like that at all’ so I am a little bit sceptical…There are. As I said, I mean in research that I’ve done there are variables and sometimes you can’t take all those varieties out. You can try but, yeah…” Joanne, Established working class

Notable, however, is that in Joanne’s quote, is the use of the term ‘sceptical’ again suggesting that working class may, more so than middle or upper class, come from a default position of distrust. This was observed among largely among working class participants.

Regardless of social class, most women demonstrated an appreciation for, or awareness of, how the scientific community evaluates quality of information. Words or phrases used to describe trustworthy information included; “evidence based” (e.g. Tricia, Established working class; Stephanie, Mobile middle class), based in evidence, rather than fads (e.g. Isabelle, Established working class), “reputable” (e.g. Isabelle, Established working class; Edith, Established middle class; Rachael, Emerging affluent class; Danielle, Established working class), be based in the “latest research” (e.g. Lesley, Established working class) provided by “experts” (Gillian, Established affluent). Indeed, women spoke to wanting access to scientific information before making decisions that might impact health:

“I trust on the whole, as a generalisation. Because I have a degree in health science, I’m well aware of the methods behind research and things like that. Once again, that puts me in a privilege position.” Joy, Established working class

“Rather than just the nice little leaflets that they give you it’s like ‘can I have the scientific papers please; I want to go and read them?’ I’m very much like that and I’m lucky because I understand them. Some people, if you haven’t had exposure to them you just look at them and they’re gobbledygook, you know.” Rebecca, Established middle class

“It’s got to be evidence-based because that’s what I do every day so that’s really important to me; that might not be important to other people.” Tricia, Established working class

Although some women looked to signals of trustworthiness based on the language of science, others were swayed by the believability of the person conveying the message. This was particularly prevalent among middle class participants who endorsed a source as being trustworthy if they were affiliated with trusted social institutions (government) or were experts with credentials (e.g., nutritionists).

Edith, Established middle class: I’d get on the internet and do a bit of a Google search and look for some reputable sources to back it up.

*Interviewer: How would you decide what a reputable source is?*

Edith, Established middle class: If it’s government-based or endorsed or hospital-based, at least then you know that they’re all evidence-based sites. I’d look at the references for who did the research and how big the sample was.

Eve (Mobile middle class) too spoke of the need for her to know *who* has written the article before she can begin to decide whether or not to trust the information.

“Yeah, in the *Sunday Mail* [a tabloid newspaper and the mainstream published Sunday newspaper in South Australia] and it never really says who writes those articles so I don’t know where the information’s come from. I would definitely Google the information from, say, magazines like that but the health magazine that I read is called–I think it’s just called Health and a lot of those articles actually are from nutritionists and things like that. I often read who’s written the article before I decide whether I can trust it or not.” Eve, Mobile middle class

However, Eve (Mobile middle class) also noted that beyond knowing their title, she would not go further to evaluate the information provided.

“Yeah, I probably just trust that if they’re a nutritionist they’ve studied to become a nutritionist. Yeah, no, I probably would just trust that their information is gospel, I guess.” Eve, Mobile middle class

Both the language of the message and its source helped interviewees determine credibility and, consequently, trustworthiness. There was some evidence that this judgement may occur differently for women in different social classes.

## Discussion

The goal of the present work was to extend our understanding of how public health communication efforts might reflect class-based differences as an avenue for working towards more equitable breast cancer prevention efforts. We present our discussion in a manner that speaks to the two proposed aims of the project: two key aims: (1) to identify in whom and in what women trust about breast cancer risk (in particular, alcohol as a modifiable risk factor) and how they determine the trustworthiness of these sources; and (2) to identify how the patterning of processes women use to make decisions about what is trustworthy is ‘classed’.

### Sources of information women (do not) trust

Women we interviewed spoke at length about not trusting information that appeared to conflict with their own interests. This finding suggests that women have learned, whether through formal education or experience, to be cautious of information gained through media, either traditional or social. Media literacy has become of increasing important with the growth of the Internet in particular, because people are now exposed to large amounts of information, which need to be sifted and evaluated [73]. The women interviewed were wary of information that appeared to serve a particular organizational or individual agenda. For example, criticisms of big business (e.g., “Big Pharma” and Monsanto) led individuals to question the interests being served in health promotion campaigns, as has been documented with other health promotion efforts (e.g. vaccine hesitancy [74]). The women we interviewed wanted transparency with regards to the intention and the origins of messages. Organisations producing breast cancer risk message(s) were trusted if they were seen to have the public’s interest at heart, a documented strategy for building trust in organisations [75]. Information was trusted particularly when the message might be to the short-term detriment of the messenger (e.g., the government losing tax revenue in promoting reduced consumption of alcohol). Interestingly, when women considered whose interests are being served by risk messaging and used this to assess the trustworthiness of information, they did not specifically identify the alcohol industry as being one of the vested interests. This is despite the real possibility that they may have seen information about alcohol and cancer delivered from Alcohol industry-funded sources such as DrinkWise Australia.

In discussing their logic for trusting, or their assessment of whether information is trustworthy, many women spoke of signals or markers of trust. Women trusted information on the basis of the organisation providing the message [76]. In this case, trust in the institution–for example, the Cancer Council of South Australia—provided a catalyst for trusting the information presented. Trust in institutions was also more subtly referred to when women explained their ease in trusting information rooted in ‘science’ and ‘research’. Here, trustworthiness was signalled when messages contained scientific terminology; which while demonstrating critical consideration of the message, may also lead women to accept messages that intentionally engage in such language to push their own agenda. For example, Hopf, Krief [77] commented, “Fakery affects science and social information and the two have become highly interactive globally, undermining trust in science and the capacity of individuals and society to make evidence-informed choices, including on life-or-death issues” (p. 1) [77]. Research regarding how we might best educate the public regarding the specific aspects of “scientific” or research-based messages that should either generate acceptance or flag the need for closer scrutiny. We need to better understand the trusted nature of scientific terminology and what that means for manipulation, misinformation, and disinformation.

Women, notably from middle and affluent classes, also spoke of their engagement with ‘common sense’. Common sense is based on lay understandings of health and illness, and was used as a ‘decision-making heuristic’ (see Ross [78] by women to accept or reject information. As a type of informal knowledge, common sense is shared among groups who subscribe to similar values and have access to similar resources. The use of ‘common sense’ by women in middle/affluent classes, and not working class, suggests that common sense may be more complex and multidimensional that previous understandings have alluded. For example, in Australia, the Cancer Council and Breast Cancer awareness campaigns have for decades promoted breast cancer awareness (including the link to alcohol consumption). For middle/affluent participants, pre-existing high levels of capital–the education, the social networks, the financial resources to be health literate, exposure to such messaging because of values and social grouping–may have allowed them to engage with ‘common sense’ as a process for assessing trustworthiness. However, differing levels of capital for other social groups might lead to differential exposure to such messaging, interpretation of the messaging and the extent to which the message becomes ingrained as ‘common sense’. It is therefore critical that we engage in approaches to messaging that are salient for women in different social groupings and identify the resources they might use to generate ‘common sense’ that aligns with public health messaging. Indeed, research investigating topics and sources of ‘memorable’ (which may be a proxy for common sense) breast cancer information suggest that the top sources of memorable information are found through media [46, 58], the channel largely used in these organizations in their promotional material. Unfortunately, data suggest that prevention is the least memorable topic of breast cancer information (6%), following early detection (38%), awareness (31%) and treatment messages (26%) [46]. A critical question then is how do we provide information about alcohol as a risk for breast cancer in a manner that makes it ‘memorable’, and particularly to the extent that it may be internalised and reproduced as part of women’s classed dispositions and therefore drive ‘common sense’? A critical part of the limitations of ‘common sense’ in women’s understandings that alcohol causes breast cancer is that women do not typically think of alcohol when they hear of a breast cancer diagnosis (unlike how we might think of smoking when we hear of a lung cancer diagnosis). Perhaps breast cancer prevention efforts, especially in so far as alcohol consumption is concerned, need to also recognise the classed limitations of common sense (is it really ‘common’?) and the assumptions entwined in understandings about health information and risk behaviours (how ‘common’ are they?). Our classed findings suggest markers of trust in breast cancer risk messaging hark back to culturally shared understandings of who and what is trustworthy–and this is cognitively embodied as common sense.

### The classed nature of trust in health information

Women in higher social class positions were generally more critical of health messages–looking to evaluate an evidence-base behind all information. They were more likely than women in working class positions to describe heuristics for determining the trustworthiness of information by questioning the validity of the source, or to make an informed judgement based on scientific evidence or rigour. Like explanations regarding the use of ‘common sense’, this may result from their access to various forms of capital that have provided a means of questioning, critically evaluating and drawing on well informed heuristics. This finding may also relate to our data suggesting that working class women start from a default position of distrust—evident in their discussion of ‘scepticism’ or taking things with a ‘pinch of salt’—in social institutions that have been structured in a way that advantages those already living with privilege. Regardless of explanation, the classed nature of our findings warrants further investigation to inform public health efforts towards cancer prevention. Consideration of the how various forms of capital shape access to and use of trustworthy health information can be used to developed tailored messaging considering class, creating more equitable provision of information.

While we sought to explore what and whom women might trust, we are cognisant that even if women trust the message and messenger, it might not lead to the desired behaviour change (as guided by public health) of reduced or null consumption. For example, in a 2016 study of responses of the Australian public to proposed cancer warning labels on alcohol products, Miller, Ramsay [79] found that many respondents agreed that labels would raise awareness but few believed this awareness would change drinking practices. Thus, we appreciate that knowing what/whom women of various social classes might trust regarding the risks of alcohol for breast cancer, might not lead to behaviour change. It is also important to note that these data are not generalisable to other alcohol-related harms (e.g., associations with other cancers) as health behaviour is shaped by a myriad of factors (e.g., knowledge, emotions [80]) that will likely be unique to the health/disease risk under consideration. Nonetheless, our work allows us to identify the best means for communicating with women, providing insight into the best heuristics for providing information to women and recognising it still may be difficult for them to reduce alcohol without additional cultural/societal shifts [12, 58]. While we did not explore trust in the messages about alcohol risks that are communicated by alcohol industry-funded organizations, this is an area for research extension. Previous research has shown the heuristics used in industry communication such as promoting ‘responsible’ drinking confuses public health messages and might have important implications for breast cancer prevention [81].

### Conclusion

Communication about alcohol as a risk factor for breast cancer is a public health priority [11]. The present work highlights class-based differences in how midlife women come to determine if information is trustworthy and thus, whether to consider it in guiding alcohol consumption as it relates to breast cancer risk. While our findings need further support through more targeted research, we suggest that some segments of the population come from a position of privilege, drawing on common sense heuristics to guide behaviour, while others may come from a default position of risk and distrust, remaining sceptical until we can demonstrate–as ‘public health’–our trustworthiness. By applying a relational approach to trust, we identify that more equitable provision of cancer prevention messaging might consider how social class shapes the reception and acceptance of risk information. Class should be considered in the development and tailoring of messages as the trustworthiness of organizations behind public health messaging cannot be assumed. Our data provide further evidence for systemic change that promotes more equitable access to forms of capital that provide a means of questioning, critically evaluating and drawing on well informed heuristics for health decisions more broadly. The fact that health detection and prevention behaviours are class-based (with lower classes fairly more poorly) [19], points to the need for change beyond the provision of tailored messaging.
